# Gender and income disparities in World Psychiatry Congress participation (2023–2024)

**DOI:** 10.1007/s00737-025-01646-2

**Published:** 2026-01-07

**Authors:** Imran Gokcen Yilmaz-Karaman, Mariana Pinto da Costa, Betul Koseoglu, Asli Ugur Oktar, Florence Thibaut, Ann Færden

**Affiliations:** 1 Department of Psychiatry, Faculty of Medicine, Eskişehir Osmangazi University, Eskişehir, Turkey; 2 Institute of Psychiatry, Psychology & Neuroscience, King’s College London, London, UK; 3 Department of Psychiatry and Addictive Disorders, INSERM U1266 Institute of Psychiatry and Neurosciences, University Paris Cité, University Hospital Cochin, Paris, France; 4 Private practice, Oslo, Norway

**Keywords:** Psychiatry, Congress, Gender, Sexism, Developing countries, Equality

## Abstract

**Purpose:**

Although the number of women in psychiatry has increased substantially, gender disparities remain in leadership and visibility at scientific meetings. Country income level also affects academic participation, but its impact within the field of psychiatry remains underinvestigated. This study examined gender and income disparities at the country level, as well as gender and income disparities within countries, at two consecutive World Congresses of Psychiatry (WCPs), held in Austria (2023) and Mexico (2024).

**Methods:**

The scientific programs of WCP 2023 and WCP 2024 were systematically reviewed to identify all speakers and chairs. Data were extracted on gender, role, session type, and country income level, classified according to World Bank criteria. Gender was determined from congress profiles, photographs, pronouns, or the Gender API. Statistical analyses included chi-square tests with Bonferroni corrections, with significance set at *p* < 0.05.

**Results:**

WCP 2023 featured 999 speakers/chairs, and WCP 2024 featured 574. Women’s representation increased significantly from 37.4% in 2023 to 43.4% in 2024 (*χ²* = 5.382, *df* = 1, *p* = 0.020). Participation from low- and middle-income countries also rose in 2024, while men’s representation from high-income countries declined. Several session types in 2024 reached or exceeded gender parity, including Distinguished Lectures (58.3%), Panel Discussions (50%), and Early Career Psychiatrist Sessions (60%).

**Conclusions:**

Women’s representation at WCPs has shown encouraging improvement, although parity has not yet been achieved, and differences are evident by country income level. Hosting congresses in middle-income countries may support broader participation. Continued monitoring, mentorship initiatives, and inclusive conference policies can further strengthen gender equality and global representation in psychiatry.

## Highlights

Women’s participation in the World Psychiatry Congress increased from 2023 to 2024.

More speakers came from low- and middle-income countries in 2024.

Hosting events in middle-income countries may boost global inclusion.

Continued efforts can strengthen women’s leadership in psychiatry.

## Introduction

The late 19th century marked a turning point in women’s access to medical education. By the 20th century, the number of women physicians and psychiatrists had risen substantially. For example, the proportion of women completing certification with the American Board of Psychiatry and Neurology increased from 5% in 1950 to 36% in 1990 (Hirshbein 2004). In the 21st century, psychiatry has become one of the most popular specialties among women. Nonetheless, women remain underrepresented in leadership roles within the field (McCarthy 2015).

This underrepresentation is often described as the “glass ceiling effect”, referring to the invisible barriers that women encounter in professional environments (Yilmaz-Karaman et al. 2022). Studies in Europe have highlighted several contributing factors in psychiatry, including gender gap in recruitment opportunities, limited access to mentorship and leadership development programs, hostile work environments characterized by sexism, restricted networking opportunities, and the disproportionate burden of time-consuming responsibilities both at work and home (Kilic et al. 2023).

In medical professions and professional organisations, leadership positions should reflect the overall proportion of women in the field (Hüsch et al. 2022). Persistent underrepresentation perpetuates gender bias and reduces the availability of women role models and mentors, thereby limiting progress towards equality (Ruzycki et al. 2019). Increasing women’s participation in scientific meetings is crucial for visibility and professional development. Such representation also provides trainees and early-career colleagues with role models (Ruzycki et al. 2019) while helping to reduce perceptions of sexism at conferences (Hüsch et al. 2022).

Achieving gender equality has become a priority in medicine in the 21st century, aligning with the United Nations Sustainable Development Goal 5.5, which states: “Ensure women’s full and effective participation and equal opportunities for leadership at all levels of decision making in political, economic and public life” (United Nations 2015). This extends to women’s participation in academic and professional forums, including scientific conferences and meetings.

The World Psychiatric Association (WPA), founded in 1950, represents over 250,000 psychiatrists from 121 countries and comprises 66 scientific sections dedicated to educational activities, networking, and research (WPA n.d.). The World Congresses of Psychiatry (WCP), serves as major international platforms for knowledge exchange and collaboration. WPA Global Survey on Psychiatry 2019 revealed that, globally 46% of psychiatrists and 54% of psychiatry trainees are women among WPA member societies (Castaldelli-Maia et al. 2025). However, equal numbers of members do not necessarily indicate the absence of a glass ceiling effect. Monitoring gender representation in visible leadership roles, such as chairs and speakers at scientific meetings, is an important component of addressing inequality (Yilmaz-Karaman et al. 2022).

This study led by the Women’s Mental Health Section, aimed to investigate the gender and country income disparities in two consecutive WCPs: Vienna in 2023 *(hosted in a high-income European country)*, and Mexico City in 2024 *(hosted in a middle-income North American country).*

## Methods

We analysed the scientific programs of the World Congress of Psychiatry (WCP) held in Vienna in 2023 and in Mexico City in 2024. The programs were obtained from the official congress websites on 09/09/2024 (https://cslide.ctimeetingtech.com/wcp23/attendee/confcal and https://cslide.ctimeetingtech.com/wcp24/attendee/confcal). For each listed participant, we collected data on gender, role (chair or speaker), session type, and country income level (classified according to World Bank standards).

The present study focused on “gender” instead of “sex” . Gender was determined from the congress profile, which typically included pictures, brief biographies, and pronouns. Where this information was unavailable (*n* = 2), the Gender API software (www.gender-api.com/) was used to identify gender based on names across cultures.

Session types were categorised according to the definitions provided in the program. Each speaker or chair was assigned a country classification based on the World Bank income level of their country of affiliation.

The inclusion criteria required participants to be listed in the program as chair, speaker, or both.

Data extraction was conducted independently by two researchers. A third researcher verified accuracy by re-coding a randomly selected 5% of entries with assistance from two other researchers, achieving a 100% agreement rate.

### Ethics

The study was approved by the Non-Invasive Clinical Research Ethics Committee of Eskişehir Osmangazi University in Turkiye on July 23, 2024, under decision number 34.

### Statistical analysis

Statistical analysis was conducted using IBM SPSS Statistics version 25. All data were categorical and presented as frequencies and percentages. Comparisons were made using the chi-square test, and post hoc analyses were performed using the Bonferroni correction. A statistically significant p-value was set at 0.05.

## Results

WCP 2023 featured 999 speakers, while WCP 2024 included 574. For each congress, one speaker’s country was missing (*n* = 998, *n* = 573). Table 1 shows the gender distribution of the speakers in WCP 2023 and WCP 2024. Women’s representation increased significantly from 37.4% in 2023 to 43.4% in 2024 (*χ²=*5.382, *df* = 1, *p* = 0.020).

**Table 1:** Gender distribution of the speakers in WCP 2023 and WCP 2024

Gender (Speakers and or chairs)	WCP 2023		WCP 2024	
	*n*	%	*n*	%
Women	374	37.4	249	43.4
Men	625	62.6	325	56.6

The two congresses differed significantly in overall gender representation (*χ²* = 63.508, *df* = 3, *p* < 0.001). Among participants from low- and middle-income countries, the proportions of both women and men increased in WCP 2024 compared with WCP 2023 (each *p* < 0.001, see Table 2). In contrast, the proportion of men speakers from high-income countries decreased in WCP 2024 (*p* < 0.001), while the proportion of women speakers from high-income countries remained stable.

**Table 2:** Gender and country income level in WCP 2023 and WCP 2024

Gender & Affiliation country (Speakers and chairs)	WCP 2023 (a)		WCP 2024 (b)		
	*n*	%	*n*	%	
Women & low-middle income	104	10.4	98	17.1	a < b*
Women & high income	270	27.1	150	26.2	-
Men & low-middle income	143	14.3	147	25.7	a < b*
Men & high income	481	48.2	178	31.1	a > b*

* : *p* < 0.001; *WCP* World Congress of Psychiatry

Table 3 presents the frequencies and percentages of speakers and chairs across session types. In WCP 2024, some formats achieved or exceeded gender parity, including the WPA Distinguished Lectures (58.3%), Panel Discussions (50%), and Early Career Psychiatrists Session (60%). WCP 2024 had more contributions from women. Other session types, however, did not reach 50% women’s participation.

**Table 3:** Session types and women’s participation in WCP 2023 and WCP 2024 as speakers and / or chairs

Session types (Speakers and chairs)	WCP 2023			WCP 2024		
	Total *n*	Women *n*	Women %	Total *n*	Women *n*	Women %
Plenary / Presidential session	44	11	25	39	17	43.6
Courses	45	22	48.9	43	11	25.6
Special session	14	2	21.4	-	-	-
Action plan sessions	29	3	10.3	-	-	-
Free communications session	87	38	43.7	87	37	42.5
Original sessions	76	34	44.7	32	14	43.8
State of the art symposia	58	12	20.7	37	15	40.5
Treatment updates sessions	33	7	21.2	-	-	-
Accepted symposium	403	169	41.9	221	108	48.9
WPA distinguished lectures	18	4	22.2	12	7	**58.3**
Panel discussions	56	23	41.1	28	14	**50**
Local language session	85	37	43.5	53	18	34
Interorganizational symposia	24	5	20.8	-	-	-
Company-supported sessions	7	1	14.3	11	4	36.4
Psychiatry worldwide	20	5	25	-	-	-
Early career psychiatrists session	-	-	-	5	3	**60**
Meet the experts	-	-	-	6	1	16.7
Total	999	374	37.4	574	249	43.4

*WCP* World Congress of Psychiatry; *WPA* World Psychiatric Association

## Discussion

### Key findings

Women accounted for 37.4% of speakers and chairs at WCP 2023, rising to 43.4% at WCP 2024. Although this increase is encouraging, both figures remain below parity. Only certain session types at WCP 2024 exceeded 50% women’s participation, including WPA Distinguished Lectures, Panel Discussions, and Early Career Psychiatrists Sessions. Notably, WCP 2024, hosted in a middle-income country, demonstrated a higher representation of both women and speakers from low- to middle-income countries. In contrast, WCP 2023, hosted in a high-income country, demonstrated a higher gender gap, with almost half (48.2%) of all speakers and chairs being men from high-income countries.

One might expect the gender gap in academic psychiatry to be smaller in high-income countries, given the generally stronger gender equality scores in these settings (United Nations Development Programme n.d.). However, our findings did not align with this expectation. The WCP 2023 in Austria attracted predominantly speakers from high-income countries. In contrast, the WCP 2024 in Mexico drew a broader mix, including more representation from speakers from low- and middle-income countries. Similar patterns have been observed at other medical and global health congresses, where geographical location and economic context of the host country shape attendance (Ruzycki et al. 2019). Barriers such as travel costs, visa restrictions, and limited institutional support tend to disproportionately affect colleagues from low- and middle-income settings, making participation more feasible when congresses are held in countries with comparable contexts or greater regional accessibility (Hüsch et al. 2022). These findings highlight not only the gender gap but also the structural inequities related to the global distribution of scientific meetings.

In addition, the increase in representation at WCP 2024 may also reflect WPA’s recent initiatives. The WPA has established an Equality, Development, Inclusion, and Transcultural Perspectives Presidential Advisory Group, led by Prof. Danuta Wasserman, following the WPA Congress in Vienna in 2023 (Wasserman 2024). The WPA Action Plan 2023-2026 aspires to address the diverse needs of individuals across the lifespan and from various cultural backgrounds, including an emphasis on gender equality (Wasserman 2024). These measures may already be contributing to progress in gender balance among WCP speakers and chairs.

### Comparison with other literature

Our findings are broadly consistent with previous research on gender representation at scientific meetings. A previous study focusing on American Psychiatric Association (APA) annual meetings reported that women accounted for 36.3% in 2009, increasing to 45.6% in 2019 (Sebbane et al. 2022). Another study from European Rheumatology conferences reported women’s representation as speakers between 39.7% and 43.5% (Hassan et al. 2022). These figures are encouraging but still fall short of gender parity.

Research from India focused on a decade-long audit of gender representation at the local rheumatology conferences, revealing that women accounted for 20% of speakers and 16% of chairs (Mohansundaram et al. 2021). A comprehensive review of gender representation at 13 major gastroenterology, hepatology, and endoscopy conferences held across the Asia–Pacific region, found that women’s overall representation as speakers was 18.4%, and the proportion of women chairs was 6.7% in 2022 (Devi et al. 2025). A cross-sectional study analyzed 12 academic psychiatry institutions worldwide to assess the representation of women at different career stages, revealing substantial regional variations. The study found that Nigeria demonstrated one of the lowest proportions of women in psychiatry, with only about 11% of faculty being women (Kenney et al. 2022). That indicates significant regional differences in the gender gap.

Surgical branches have even higher gender gaps. For example, the percentage of women among speakers in ophthalmological conferences was reported as 25% (Arslan et al. 2025), and only 8.3% of first authors at neurosurgical conferences were women (Johnson et al. 2022). These disparities may reflect stronger competition, which may exacerbate barriers to women’s advancement (Yilmaz-Karaman et al. 2022).

Barriers to equitable participation in professional development and international scientific meetings are closely linked to gender and structural inequalities within psychiatry. A survey conducted in Europe identified several key obstacles, including gender discrimination, limited institutional support, and insufficient access to mentorship and leadership opportunities (Kilic et al. 2023). Women psychiatrists, in particular, reported lower confidence in self-promotion and networking, as well as greater exposure to implicit bias, which collectively restricts their visibility and advancement (Kilic et al. 2023). These barriers highlight the need for targeted strategies to ensure fair representation and career progression in academic psychiatry.

“Conference equity” has been described as the term for the underrepresentation of researchers from low- and middle-income countries (Velin et al. 2021). Researchers from low-middle-income countries face barriers, including limited speaking opportunities, financial barriers, visa restrictions, political barriers, and racism and discrimination. Measures such as relocating conferences to low- and middle-income countries, providing scholarships, and offering administrative and advocacy support, as well as research empowerment, have been shown to facilitate participation (Velin et al. 2021).

### Strengths and limitations

This study has several strengths. To our knowledge, it is the first to compare gender and country income disparities across two consecutive WPA Congresses held in countries with different income levels. The large number of speakers and chairs provides a comprehensive overview, while the rigorous data verification process, including independent double coding and independent random re-checking of the dataset with 100% concordance, increases the reliability of the findings.

Limitations must also be acknowledged. Gender identification relied mainly on online profiles, pronouns and names, which may not align with the speaker’s or chair’s identified gender; and the use of the Gender API software might have led to misclassification in two cases where gender was uncertain. The analysis was limited to two congresses, which restricted its ability to capture longer-term trends. Other dimensions of diversity, such as ethnicity, career stage, or seniority, were not assessed. Finally, while participation was examined by country income level, the analysis could not fully account for structural barriers, such as funding, visa restrictions, or institutional support, that may affect attendance particularly from low- and middle-income countries.

### Implications for future practice and research

Mentorship remains a pivotal factor in promoting gender equality in psychiatry. Future research should explore structured mentorship programs that actively support women and colleagues from low- and middle-income countries in gaining visibility at international congresses. Studies could explore how mentorship networks influence career advancement, conference participation, and leadership opportunities, with a particular focus on the role of women mentors and role models. Comparative studies across disciplines may also help to identify best practices in mentorship and professional development that could be adapted for psychiatry. Longitudinal research further clarifies whether sustained mentorship initiatives contribute to narrowing gaps in academic visibility and leadership over time.

Practical measures can also strengthen monitoring and accountability. One such measure is the collection of self-reported gender data during the submission of abstracts or symposia. As highlighted by Hassan et al. (2022), reliable gender-disaggregated data are essential for evaluating equality in academic conferences. Current practices often rely on assumptions based on names or photographs, which are prone to error and may fail to capture non-binary identities. Introducing an optional gender field in submission systems would allow congress organizers to collect self-reported data. Such data would also facilitate longitudinal monitoring of progress toward gender equality across different congresses and settings.

## Conclusions

This study found that women’s representation as speakers and chairs at the WCPs improved between 2023 and 2024, although full parity has not yet been reached. Hosting the congress in middle-income countries was associated with greater representation from low- and middle-income regions, suggesting that the location of congresses may influence inclusivity.

These findings highlight the intersection of gender and country income level related inequalities in international psychiatry meetings. Ongoing efforts by the WPA, including its Equality, Development, Inclusion, and Transcultural Perspectives Advisory Group, provide an important foundation for addressing these challenges. Further progress will depend on sustained monitoring, mentorship, equitable funding mechanisms, and inclusive conference policies. Future research should investigate multiple congresses and disciplines to assess trends, evaluate progress, and identify effective strategies for achieving equality in academic psychiatry worldwide.

## Data Availability

No datasets were generated or analysed during the current study.
